# Molecular characterization of thyroid hormone receptor beta from *Schistosoma japonicum* and assessment of its potential as a vaccine candidate antigen against schistosomiasis in BALB/c mice

**DOI:** 10.1186/1756-3305-5-172

**Published:** 2012-08-13

**Authors:** Chunhui Qiu, Shengfa Liu, Yang Hong, Zhiqiang Fu, Meimei Wei, Dezhou Ai, Jiaojiao Lin

**Affiliations:** 1 School of Life Sciences, Xiamen University, Xiamen, 361005, Fujian Province, China; 2 Shanghai Veterinary Research Institute, Chinese Academy of Agricultural Sciences, Key Laboratory of Animal Parasitology, Ministry of Agriculture of China, Shanghai, 200241, P R China

## Abstract

**Background:**

Thyroid hormones (TH) modulate growth, development and differentiation and metabolic processes by interacting with thyroid hormone receptors (THRs). The purpose of this study was to identify a novel thyroid hormone receptor beta encoding gene of *Schistosoma japonicum* (SjTHRβ) and to investigate its potential as a vaccine candidate antigen against schistosomiasis in BALB/c mice.

**Methods:**

The full-length cDNA sequence of SjTHRβ, its gene organization, and its transcript levels were characterized, and the phylogenetic relationship between THR, RAR and RXR from other organisms were analysis, the ability of this protein binding to a conserved DNA core motif, and its potential as a vaccine candidate antigen against schistosomiasis in BALB/c mice were evaluated.

**Results:**

The SjTHRβ cDNA was cloned, verified by 5’ and 3’ Rapid Amplification of cDNA Ends and shown to be polyadenylated at the 3’end, suggesting the transcript is full-length. SjTHRβ is homologous to THRs from other species and has a predicted conservative DNA binding domain and ligand binding domain that normally characterizes these receptors. A comparative quantitative PCR analysis showed that SjTHRβ was the highest expressed in 21d worms and the lowest in 7 d and 13 d schistosomula. The cDNA corresponding to DNA binding domain (SjTHRβ-DBD) and ligand binding domain (SjTHRβ-LBD) were cloned and subsequently expressed in *E coli*. The expressed proteins were used to immunize mice and generate specific serum against recombinant SjTHRβ (rSjTHRβ). Western blotting revealed that anti-rSjTHRβ-LBD serum recognized two protein bands in extracts from 21 d worm with molecular sizes of approximately 95 kDa and 72 kDa. Electrophoretic mobility shift assay (EMSA) analysis showed that rSjTHRβ-DBD could bind to a conserved DNA core motif. Immunization of BALB/c mice with rSjTHRβ-LBD could induce partial protective efficacy(27.52% worm reduction and 29.50% liver eggs reduction)against schistosome infection. Enzyme-linked immunosorbent assay showed that mice vaccinated with recombinant SjTHRβ-LBD (rSjTHRβ-LBD) generated increased levels of specific IgG, IgG1 and IgG2a antibody. Bio-plex analysis demonstrated that rSjTHRβ-LBD induced considerably higher levels of T helper 1 cytokines (IL-2, IL-12 and TNF-α) than T helper 2 cytokines (IL-10, IL-4), suggesting that rSjTHRβ-LBD vaccination could stimulate mixed Th1/Th2 types with Th1 dominant immune responses.

**Conclusions:**

Our study presented here identified SjTHRβ as a new schistosome THR that might play an important role in host-parasite interaction and be a vaccine candidate for schistosomiasis.

## Background

Thyroid hormones (TH) modulate growth, development and differentiation and metabolic processes by interacting with THRs [1,2]. THR which belongs to nuclear receptor (NR) superfamily 1 possesses a typical nuclear receptor modular motif, including a poorly conserved N-terminal A/B domain, a highly conservative DNA binding domain (DBD), a hinge region and a moderately conservative C-terminal ligand binding domain [2]. In the traditional model of THR action, THR regulates gene expression through its binding to the promoter region of the target gene called specific thyroid hormone- response elements (THRE) by the DBD [3]. A number of artificial and natural THREs are a direct, inverted or everted repeat or palindrome of the DNA sequence AGGTCA. THR can bind to the THRE as a monomer, a homodimer or as a heterodimer with RXR and positively or negatively control the gene transcription and expression [4].

Schistosomiasis caused by parasitic blood flukes is a major public health problem worldwide. Approximately 200 million people are infected with pathogenic schistosomes, of which 20 million have advanced to life-threatening disease [5,6].

Schistosomes live in the vascular system of the host, where they feed on blood components and respond to molecular signals from the host to develop normally [7].

Earlier researches have revealed that schistosomes can take advantage of signals from each other and from the host to facilitate their development, however, little is known about the molecular characterization of these signals [8-10]. Previous studies suggest that the existence of THR in *S. japonicum* and the parasite may use an endogenous TH/THR signalling pathway for growth and development [11]. Herein, a novel mammalian orthologue of thyroid hormone receptor beta of NR superfamily 1 from *S. japonicum* (SjTHRβ) was identified and characterized. Study of schistosome NR will help us to understand how they regulate signaling pathways in the schistosome itself and add to our knowledge of the molecular relationship between the schistosome and its hosts [12].

## Methods

### Parasites and animals

The life cycle of *S. japonicum* (Chinese mainland strain, Anhui isolate) was maintained in New Zealand rabbits and *Oncomelania hupensis* in Shanghai Veterinary Research Institute, Chinese Academy of Agricultural Sciences. Specific pathogen free (SPF) male BALB/c mice, 6 weeks old, were purchased from Shanghai laboratory animal center, Chinese academy of sciences (Shanghai). 7d, 13d, 21d, 28d, 35d and 42d Schistosomes were collected by perfusion of New Zealand rabbits infected percutaneously with 1000 to 6000 cercariae of *S. japonicum* respectively, shed from *Oncomelania hupensis* snails as described [13]. Animal care and all procedures involving animals were conducted according to the principles of the Shanghai Veterinary Research Institute, CAAS.

### Cloning of *S. japonicum* thyroid hormone receptor

The total RNAs were extracted from 21d worms using Trizol reagent (Invitrogen), following the manufacturer’s instructions. DNA remaining in the RNA solution was digested with DNase I (TaKaRa) and then purified by an RNeasy mini kit (Qiagen, Germany). The concentration and purity of the RNAs were evaluated by spectrophotometry (Eppendorf, German). The cDNAs were synthesized using the PrimeScriptTM RT reagent Kit (TaKaRa, China), according to the user manual. According to putative thyroid hormone receptor(predicted Sjc_0027230) sequence deposited in http://lifecenter.sgst.cn/schistosoma/cn/schdownload several pairs of primer were designed. Two correct PCR products corresponding to partial fragment of putative thyroid hormone receptor were amplified individually by primer1 (Forward primer: 5^′^-AACAAACTACGAACGCAAAG-3^′^ and reverse primer: 5^′^-TTAAAGCTACCGCACGAA-3^′^) and primer2 (Forward primer: 5^′^-ATTATCGTGCTATGACTTG-3^′^ and reverse primer: 5^′^-GTCTTTGCGTTCGTAGTT-3^′^). In order to obtain complete mRNA sequence, two pairs of RACE primers were designed based on amplified PCR sequence. After total RNAs were extracted, reverse transcription (RT) and PCR were performed according to the user’s manual of the SMART^TM^ RACE cDNA Amplification Kit (Clontech). The transcription initiation site and the 3′-end of SjTHRβ were amplified by 5^′^-RACE and 3^′^-RACE, respectively. The 3^′^-RACE was performed with the following primers: forward gene-specific primer, 5^′^-TCACTTGGAATCGTCGAACCTA-3^′^; forward gene-specific nested primer, 5^′^-GCCCAAATGATTCGTGCGGTAG-3^′^. The 5^′^-RACE was performed with the following primers: reverse gene-specific primer, 5^′^-CCATCCCACCGCTTATGCACCGATCAAA-3^′^; reverse gene-specific nested primer, 5^′^-GCTTATCAGAAACAGAGCAGCGACCTTG-3^′^; PCR products obtained from RACE were ligated with pMD19-T vector (TaKaRa) and were then transformed into DH5α competent cells (Invitrogen). The positive clones were detected and sequenced. DNAStar software was employed to assemble the sequences of 3^′^-RACE, 5^′^-RACE, and mRNA into a complete cDNA sequence.

### Real-time PCR analysis

The mRNA expression of SjTHRβ was evaluated in 6 different developmental stages including 7, 13, 21, 28, 35 and 42 d worms of *S. japonicum*. The cDNA products were obtained by the method described above. The primers specific to SjTHRβ (forward: 5^′^-AGTTCCTGAATTTGAGCTGT-3^′^ and reverse: 5^′^-TTTCTTGTTGATGAGACGGC-3^′^) can generate an amplicon of 218 bp. *S*. *japonicum* NADH-ubiquinone reductase was used as a housekeeping gene for this study [14]. Primers specific for NADH-ubiquinone reductase gene were 5^′^-CGAGGACCTAACAGCAGAGG-3^′^ (sense) and 5^′^-TCCGAACGAACT TTGAATCC-3^′^ (antisense), and the PCR product size of the internal control is 174 bp. The PCR amplification was carried out using the reverse-transcribed cDNA as template with the SYBR Premix Ex Taq^TM^ (TaKaRa, Japan) using the Mastercycler ep realplex4 (Eppendorf, Germany) real-time PCR detection System. The cycling protocol was as follows: 95°C for 10 s and 40 cycles of 95°C for 15 s, 55°C for 15 s, 72°C for 15 s. The value of fluorescence was detected at the end of each extension step. Specificity of the PCR products was determined by melting curve analysis and agarose gel electrophoresis. Negative control reactions, which contain all of the reagents except cDNA template, were included to make sure that the reaction system was not contaminated. Each reaction was performed in triplicate the entire experiment was carried out at least three times.

### Gene organization

The web-based query system (http://lifecenter.sgst.cn/blast/cn/) was used to BLAST search the cDNA sequence. The exon/intron boundaries were determined.

### Phylogenetic analysis of SjTHRβ

Fifteen nuclear receptor protein sequences from different species were evaluated in the phylogenetic analysis. The phylogenetic tree was constructed from deduced amino acid sequences of SjTHRβ. After alignment with CLUSTAL program in Mega4, an evolutionary tree was drawn by the neighbor-joining method.

### Generation of anti rSjTHRβ-DBD and anti rSjTHRβ-LBD specific antibodies

DNA sequence encoding DNA binding domain (amino acid 119–250) of SjTHRβ was amplified by PCR using the forward primer 5^′^-GCGGAATTCCCTCCTAAAGTTAAAAAAAGAG-3^′^ and the reverse primer 5^′^-GCGCTCGAGAGTAGTCAGTGAATCTGTAAC-3^′^. The DBD fragment was then subcloned into the pET28a (+) expression vector between the restriction recognition sites of *Eco*R I (5^′^end underline) and *Xho* I (3^′^end underline). The recombinant plasmid expressed SjTHRβ-DBD in *E. coli* BL21 (DE3). LBD sequence corresponding to the predicted LBD (amino acid 556–817) of SjTHRβ was amplified by PCR using the forward primer 5^′^-GCGGGATCCGATATAGACAAAGATCCTG-3^′^ and the reverse primer 5^′^-GCGCTCGAG ATCAGTCTGCTGGATCAT-3^′^. The LBD fragment was subcloned into the pET32a (+) expression vector between the restriction recognition sites of *Bam*H I (5^′^end underline) and *Xho* I (3^′^end underline). The recombinant plasmid expressed SjTHRβ-LBD fusion protein in *E. coli* BL21 (DE3). These fusion proteins were purified by affinity chromatography on Ni^2+^-NTA resin. After dialysis against PBS, the purified fusion proteins of SjTHRβ-DBD and SjTHRβ-LBD were used to immunize BALB/c mice. The mice were injected subcutaneously four times at two week intervals with the purified protein in Montanide ISA 206 adjuvant (20 μg/100μL/mouse; Seppic). Antiserums were collected two weeks after the last injection.

### Western blot

For Western blot, 21d worm antigen was prepared from worms of *S. japonicum* and unrelated protein was extracted from *E. coli* BL21 (DE3) as described (You et al. 2010). The extracts of 21d worm and unrelated protein were separated by SDS/PAGE (12% gel) and then transferred onto nitrocellulose membrane (Whatman, Germany). The membranes were blocked with 5% non fat milk in PBS (blocking buffer) containing 0.05% Tween20 for 1 h. After washing, the membrane was incubated in a 1:100 dilution of anti SjTHRβ-LBD mouse serum overnight at 4°C. Subsequently, horseradish peroxidase conjugated goat anti-mouse secondary IgG (Sigma, USA) was used to bind first antibody. Three washes with 10mins each were performed after each step. Finally, detection was performed with the 3, 3^′^5, 5^′^-Tetramethyl Benzidine dihydrochloride (TMB, Sigma, USA) according to the instruction of manufacturer. Image was obtained by using ImageQuant 300 Capture Imaging System (GE Healthcare, USA).

### EMSA

EMSA was performed using a LightShift chemiluminescent EMSA kit (Thermo scientific, pierce, USA) with biotin-labeled oligonucleotide (Invitrogen, China), corresponding to sequence of Half-site (5^′^-GTACCGTAAGGTCACTCGCG-3^′^) and DR0 (5^′^-CCGTAAGGTCAAGGTCACTCG-3^′^). The binding reactions were incubated on ice for 30 mins in 20 μL reaction mixture containing 2 μL 10 × binding buffer, 10 μL ultrapure water, 1 μL 50% glycerol, 1μL 100 mM MgCl_2,_ 1 μg ploy(dI: dC), 1 μL 1%NP-40, 2 μL dialyzed rSjTHRβ-DBD and 2 μL biotin-probe. After binding reaction, samples were loaded onto 6.5% polyacrylamide gel in 0.5 × Tris-borate- EDTA buffer and electrophoresed at 100 V at 4°C for 1 h. Biotin-labeled, double-stranded DNA was transferred to positively charged nylon membrane (Thermo scientific, pierce, USA). After cross-linking the transferred DNA to membrane by UV-light, biotin-labeled DNA was integrated with streptavidin-horseradish peroxidase conjugate. Finally, the result was detected using enhanced chemiluminescence and the images were taken by Image Quant LAS 4000MINI (GE, USA). For the non-specific competition experiment, the sequence (5^′^-CTAGTATACGTACTACTCTGA-3^′^) was designed and annealed to its complementary oligonucleotide and then added into the binding reaction mixture. Half-site and DR0 without biotin-label were synthesized and added into the binding reaction in the specific competition experiment. For the supershift experiment, 2 μg dialyzed rSjTHRβ-DBD was incubated with 1μL anti rSjTHRβ-DBD serum for 10 mins before incubation with the biotin-labeled probe.

### Evaluation of immune protection against *S. japonicum* challenge induced by the r SjTHRβ-LBD

Thirty-six mice were randomly allotted into 3 groups of 12 mice per group and injected subcutaneously (SC) three times at 2 week intervals with 206 adjuvant (Seppic, France) in PBS (100 μl/mouse), rSjTHRβ-LBD in 206 adjuvant (20 μg/100 μl/mouse), and PBS only (100 μl/mouse), respectively. Two weeks after the last injection, all mice were challenged with 40 ± 2 cercariae. The worm and egg counting reduction rates were used to evaluate the efficacy of immunization. Adult worms perfused from BALB/c mice were obtained and counted. The worm reduction rates were calculated using the formula: percentage reduction in worm burden = (mean worm burden of control group − mean worm burden of vaccinated group)/mean worm burden of control group × 100%. Livers of each infected mouse were weighed and homogenized in 5 ml of PBS, and then 5 ml of 10% NaOH was added and mixed. The mixture was incubated at 56°C for 45 min and then mixed sufficiently. 10 μl mixture was used to count the number of eggs and it was performed in triplicate. The data was transformed to eggs per gram (EPG). Egg counting reduction rate was calculated by the formula (mean EPG of control group – mean EPG of vaccinated group)/mean EPG of control group × 100%.

### Detection of SjTHRβ-LBD specific antibodies

Mouse sera was collected by retro-orbital bleeding before the first vaccination, 10 days post each vaccination and before the mouse perfusion. ELISA assay was performed to detect specific IgG antibodies against rSjTHRβ-LBD. rSjTHRβ-LBD at a working concentration of 10 μg/ml (pH 9.6) was coated on 96-well microtiter plates (Costar, USA) overnight at 4°C. The wells were blocked with 5% skimmed milk in PBS containing 0.05% Tween20 (PBST) for 1 h at 37°C, thereafter washed 3 times by PBST. All test sera were diluted in 1:100 in PBST, added onto plates and incubated for 1 hr. The goat anti-mouse IgG conjugated horseradish peroxidase (Sigma, USA) in 1:2500 dilutions was added to bind the primary antibody. Thorough washes were taken after each antibody incubation step. Substrate of 100 μl soluble 3,3^′^5, 5^′^-tetramethyl benzidine dihydrochloride (TMB, Sigma, USA) was added to each well. The plates were incubated at 37°C in the dark for 10 min. The reaction was terminated by 2 M sulfuric acid (50 μl/well). The absorbance was read at 450 nm on microplate reader (BioTek, USA). For IgG1 and IgG2a detection, we used goat anti-mouse IgG1 and IgG2a conjugated horseradish peroxidase (Sigma, USA) in 1:2000 dilutions as secondary antibodies, and the other steps were the same with those described in IgG detection.

### Cytokine analysis

Bio-Plex mouse cytokine assay for simultaneous quantization of interleukin (IL)-2, IL-4, IL-10, IL-12, tumor necrosis factor (TNF)-α, interferon (IFN)-γ was employed according to the recommended procedure. In brief, the premixed standards generated a stock concentration of 32,000 pg/mL for each cytokine. The standard stock was serially diluted in PBS to generate 9 points for the standard curve. The assay was performed in a 96-well filtration plate supplied with the assay kit. Premixed beads (50 μL) coated with target capture antibodies were transferred to each well of the filter plate and washed twice with Bio-Plex wash buffer. Premixed standards or anti rSjTHRβ-LBD serum (50 μL) were added to each well containing washed beads. The plate was shaken for 30 s with 1,100 rmp and then incubated at room temperature for 30 min with lower shaking speed. After incubation and 3 washes, premixed detection antibodies (50 μL) were added to each well. The incubation was terminated after shaking for 10 min at room temperature. After 3 washes, the beads were resuspended in 125 μL of Bio-Plex cytokine assay buffer. Beads were read on the Bio-Plex suspension array system, and the data was analyzed using Bio-Plex ManagerTM software with 5PL curve fitting.

### Statistical analyses

Results in this work were acquired from at least three replicates on independent experiments with identical protocols. All values are expressed as means ± SD. Statistical analyses were performed by Student's *t* test, and P < 0.05 was defined as statistical significance.

## Results

### Sequence analysis of *S. japonicum* thyroid hormone receptor

**Figure 1:**
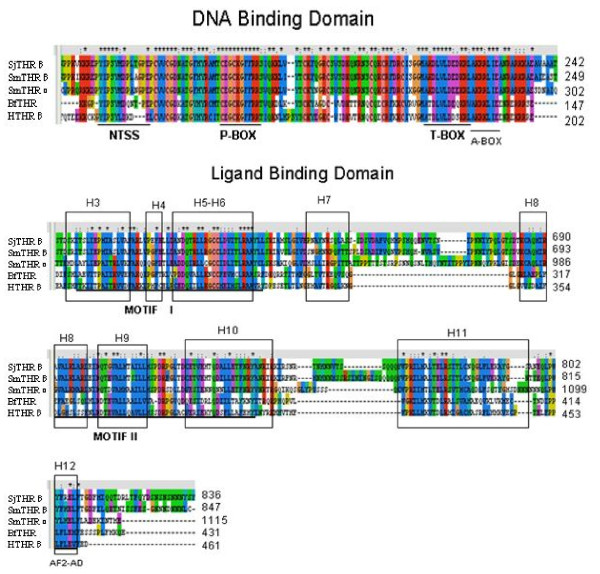
**Sequences alignment.** NTSS denotes N-terminal signature sequence. The P-Box, T-Box and A-Box are noted by single underline, respectively. Helices 3–12 within the LBD were marked by solid boxes. The Motif I and Motif II regions were underlined in the LBD. The AF2-AD in the LBD was noted by single underline. Asterisks indicate the conserved residues. The conserved residues of these sequences are highlighted with the same color. The number at the end of each line indicates the residue position in the original sequence. Sequences used in the mutiple alignment include: *Schistosoma mansoni* SmTHRβ (GenBank accession number AAR29359.1), *Schistosoma mansoni* SmTHRα (GenBank accession number XP_002573733.1), *Branchiostoma floridae* BfTHR (GenBank accession number ABS11249.1), *Homo sapiens* HTHRβ (GenBank accession number NP_001121649.1), *Schistosoma japonicum* SjTHRβ.

**Figure 2:**
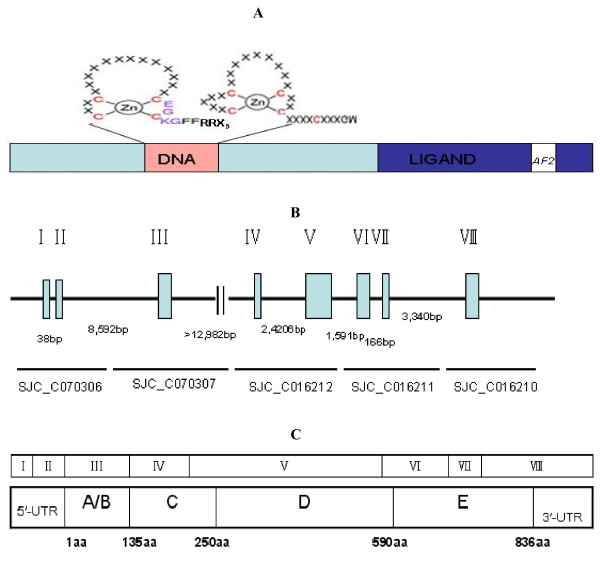
**Gene structure of SjTHRβ.A** Two zinc-finger modules of DNA Binding Domain in SjTHRβ **B** Exons (roman numerals) and introns are shown with bp sizes indicated. The five genomic supercontigs obtained from the SDSPB genomic sequence database (SJC_C070306, SJC_C070307, SJC_C016210, SJC_C016211 and SJC_C016212) are shown in the figure. Roman numerals indicate exons. **C** The amino acid size of exons and their corresponding NR domains are shown. A/B, A/B domain; C, DNA binding domain; D, hinge domain; E, ligand binding domain.

Using PCR,3^′^- RACE and 5^′^- RACE, a sequence encoding SjTHRβ from *S. japonicum* cDNA was cloned. The SjTHRβ fragment was 3196 bp in length and contained 357 bp of 5^′^-untranslated region (UTR) and 328 bp of 3^′^-UTR. The predicted entire open reading frame (ORF) of SjTHRβ includes 836 amino acids with an approximate molecular weight of 94 kDa. NCBI BLASTP search indicated that the best match for this protein sequence was *S. mansoni* THRβ (GenBank accession no. AAR29359.1) with 66% identity. In addition, SjTHRβ shows 20%, 18% and 35% identities with *Homo sapiens* THRβ (HTHRβ), *Branchiostoma floridae* THR(BfTHR) and *Schistosoma mansoni* THRα(SmTHRα), respectively. Alignment of those sequences indicated that the major functional regions were very conserved (Figure 1). So, this clone was named as *S. japonicum* THRβ (SjTHRβ). The SjTHRβ was found to possess a typical nuclear receptor modular motif, including a relatively diverse N-terminal A/B domain, a hinge region (D domain), a highly conservative DBD, and a moderately conservative C-terminal LBD. The SjTHRβ-DBD possesses two zinc-finger modules (Figure 2 A). There is an N-terminal signature sequence which goes before the first zinc-finger module (Figure 1) [2]. In the DNA binding domain, the P-box (CEGCKG) is known to play an important role in determining DNA binding specificity. The P-box in SjTHRβ is followed by a FFRR sequence, which is identical to that of retinoic acid receptors superfamily and is one of the distinguishing features of NR superfamily 1 (Figure 1) [2]. T-box which follows the second zinc finger of the DBD consists of AKDLVLDEDKRL (Figure 1). The T-box plays an important accessory role in forming the dimer interface that determines specificity for the response element recognized [15]. A-box follows the T- box, which is less conserved than either the P-box or T- box. In SjTHRβ, the A-box was AKRRLIE. The LBD of NRs is pivotal for the NR transactivation activity by providing a ligand-binding site, a major dimerization surface and a ligand dependent activation function-2 (AF2-AD) for recruiting cofactors. Like various nuclear receptors in *Schistosoma mansoni*, the helices 1–2 in the LBD of SjTHRβ are highly diverse [16]. However, helices 3–12 are relatively conserved. As shown in Figure 1, both helices 3–6 in the motif I [(F,W,Y) (A,S,I) (K,R,E,G) XXX (F,L) XX (L,V,I) XXX (D,S) (Q, K) XX (L,V) (L,I,F)], which corresponds to part of the co-activator binding surface and is the signature of the LBD (Ts), and helices 8–10 in the consensus motif II. [(EFXXXLXXLXLDXXEKAIXLFSXDRXGLXXXXXVEXLQEXXXXALXXY)] were present with high conservation in SjTHRβ [14]. In SjTHRβ, the AF2-AD core structure was identified and comprised the sequence YFRELF.

### Gene organization analysis

The cDNA sequence of SjTHRβ was queried by a BLASTN search against the published *S. japonicum* genomic supercontigs in order to elucidate the SjTHRβ gene organization. Five shotgun sequences (SJC_C070306, SJC_C070307, SJC_C016210.

SJC_C016211 and SJC_C016212), which covered the 5^′^-UTR, the entire ORF, and the 3^′^-UTR, were obtained. It was found that the SjTHRβ gene spanned more than 32 kb and consisted of eight exons (bp sizes of 153, 168, 413, 217, 1028, 359,131 and 678) and seven introns (Figure 2B). Comparison with the genomic supercontigs revealed that the A/B domain and 3^′^-UTR are encoded by one exon. 5^′^-UTR, DNA binding domain and Hinge domain of SjTHRβ protein are composed by two exons. The ligand binding domain (LBD) is encoded by three exons. The sizes of each intron were shown in Figure 2B.

### Phylogenetic analysis of SjTHRβ

**Figure 3:**
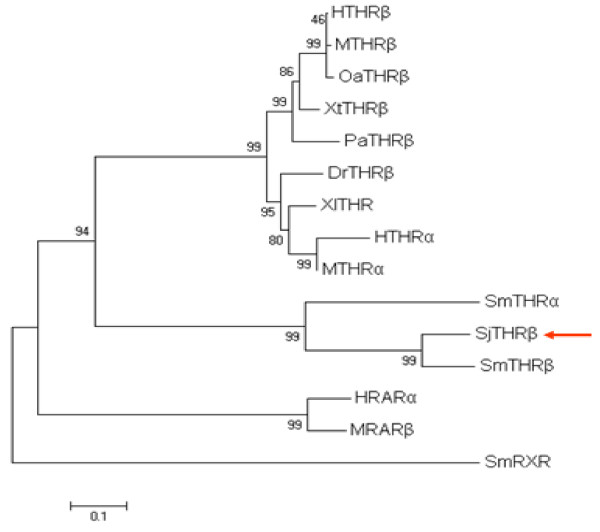
**Phylogenetic analysis of SjTHRβ.** The phylogenetic tree derived from 15 protein sequences was constructed by the Cluster W program and plotted with MEGA4. The Genbank accession numbers used in the analysis were: HTHRβ, *Homo sapiens,* NP_001121649.1; MTHRβ, *Mus musculus*, AAI19554.1; OaTHRβ, Ovis aries, NP_001177320.1; XtTHRβ, *Xenopus (Silurana) tropicalis*, NP_001039270.1; PaTHRβ, *Pseudopleuronectes americanus*, AAV66918.1; DrTHa, *Danio rerio*, NP_571471.1; XlTHR, *Xenopus laevis*, AAA49969.1; HTHRα, *Homo sapiens*, NP_001177848.1; MTHRα, *Mus musculus*, NP_835161.1; SmTHRα, *Schistosoma mansoni*, XP_002573733.1; SmTHRβ, *Schistosoma mansoni*, AAR29359.1; SjTHRβ, *Schistosoma japonicum*, HRARa, *Homo sapiens*, P10276; MRARb, *Mus musculus*, P22605; SmRXR, *schistosoma mansoni*, AF094759.

A phylogenetic tree was constructed based upon the 15 known protein sequences. HRARa, MRARb and SmRXR were used as outgroup. The analysis positioned SjTHRβ into the nuclear receptor superfamily NR1 and SjTHRβ was phylogenetically related to the thyroid hormone receptors. In addition, the SjTHRβ clustered with the SmTHRs, indicating that SjTHRβ is most closely related to SmTHRβ (Figure 3).

### mRNA expression level of SjTHRβ in the development of schistosomula into adult worms obtained from rabbit

**Figure 4:**
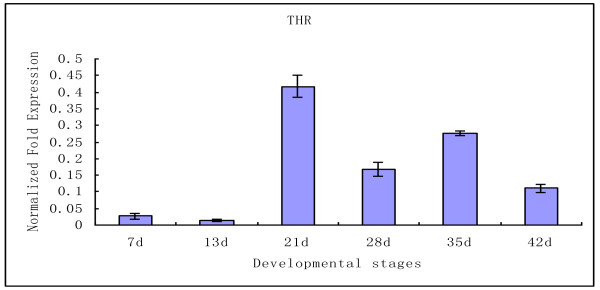
**mRNA expression of SjTHRβ in*S. japonicum*by real-time quantitative PCR.** Data were normalized against the amplified internal housekeeping control gene SjNADH. Worms were collected from infected rabbits at day (d) 7, 13, 21, 28, 35 and 42 days post-infection. This result was obtained by three separate assays with identical protocols. Vertical bars denote the ± SD about the mean.

Quantitative real-time PCR was performed to evaluate relative mRNA levels of SjTHRβ in six different developmental stages. The result revealed that SjTHRβ mRNA was expressed in 7, 13, 21, 28, 35 and 42d worms but with different levels (Figure 4). Interestingly, it showed the highest expression in 21d worms and was much lower in 7d and 13d schistosomula.

### Western blot

**Figure 5:**
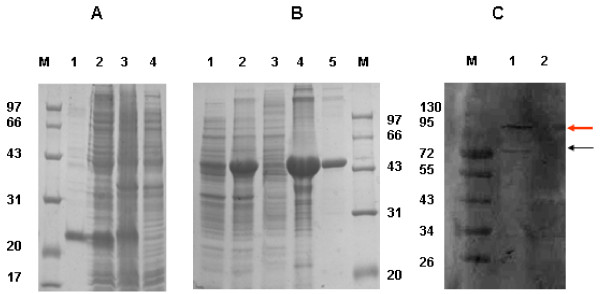
**Western blot.** ( **A**) SDS–PAGE analysis of the rSjTHRβ-DBD protein. Lane1: Purified rSjTHRβ-DBD protein (about 22 kDa). Lane2: Soluble fractions of expressed product of pET28a (+)-SjTHRβ-DBD induced with IPTG of the *E. coli* extract. Lane3: Expressed product of pET28a (+)-SjTHRβ-DBD induced with IPTG. Lane4: Expressed product of pET28a (+)-THRβ-DBD without induction. ( **B**) SDS–PAGE analysis of the rSjTHRβ-LBD protein. Lane1: Expressed product of pET32a (+)-SjTHRβ-LBD without induction. Lane2: Expressed product of pET32a (+)-SjTHRβ-LBD induced with IPTG. Lane3: Soluble fractions of expressed product of pET32a (+)-SjTHRβ-LBD induced with IPTG of the *E.coli* extract. Lane4: Insoluble fractions of expressed product of pET32a (+)-SjTHRβ-LBD induced with IPTG of the *E.coli* extract. Lane5: Purified rSjTHRβ-LBD protein (about 45 kDa) ( **C**) The protein extract of 21d schistosomes (lane1) and *E.coli* BL21 (DE3) (lane2) were probed with mouse serum specific to rSjTHRβ-LBD. The blots are representative of three replicates. M: protein marker.

The rSjTHRβ-DBD protein was purified in soluble formation with a molecular weight of about 22 kDa, compared to rSjTHRβ-LBD fusion protein in insoluble fraction with a molecular weight of around 45 kDa (Figure 5A lane1 and B lane5). Polyclonal serum against rSjTHRβ-DBD and rSjTHRβ-LBD was produced by immunizing mice. The polyclonal anti rSjTHRβ-LBD serum recognized two protein bands in extract from 21 d worms with molecular sizes of approximately 95 kDa and 72 kDa (Figure 5C lane1) and no positive band was observed in extract from *E. coli* BL21 (DE3). The band of 95 kDa is more apparent than the band of 72 kDa. Due to no evidence of two copies of SjTHRβ genes, two forms of SjTHRβ might result from alternative splicing.

### rSjTHRβ-DBD protein binds to Half-site and DR0 in vitro

**Figure 6:**
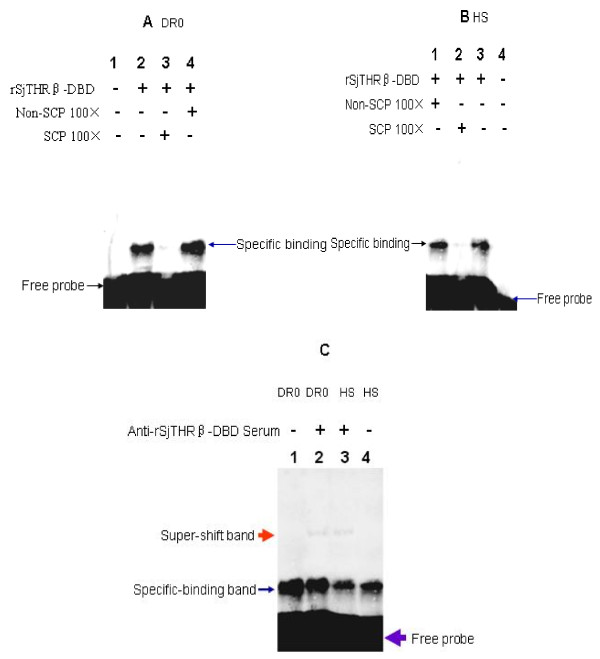
**rSjTHRβ-DBD protein binds to Half-site and DR0 in vitro*.*** ( **A**) Binding of rSjTHRβ-DBD protein to DR0. Lane1: Reaction mixture without rSjTHRβ-DBD as negative control. Lane2: Contains rSjTHRβ-DBD. Lane3: Contains rSjTHRβ-DBD with 100 fold of Specific competitive probe (SCP). Lane4: Contains rSjTHRβ-DBD with 100 fold of Non specific competitive probe (Non-SCP) ( **B**) Binding of rSjTHRβ-DBD protein to Half-site (HS). Lane1: Contains rSjTHRβ-DBD with 100 fold of Non specific competitive probe (Non-SCP). Lane2: Contains rSjTHRβ-DBD with 100 fold of Specific competitive probe (SCP). Lane3: Contains rSjTHRβ-DBD. Lane4: Reaction mixture without rSjTHRβ-DBD as negative control. ( **C**) Super-shift experiment. Lane1: Probe DR0 without anti-rSjTHRβ-DBD Serum. Lane2: Probe DR0 with anti-rSjTHRβ-DBD Serum. Lane3: Probe HS with anti-rSjTHRβ-DBD Serum. Lane4: Probe HS without anti-rSjTHRβ-DBD Serum.

rSjTHRβ-DBD protein was over-expressed in *E. coli* and purified. EMSA analysis of rSjTHRβ-DBD-DR0 and Half-site (Hs) interactions was performed in vitro. Gel shift analyses revealed that rSjTHRβ-DBD bound to DR0 and Half-site sequences. Due to DR0 containing one more AGGTCA sequence than Half-site, rSjTHRβ-DBD showed higher affinity to DR0 (Figure 6 A lane2 and B lane3). Formation of the rSjTHRβ-DBD - DR0 and Half-site complex could be competitively inhibited by addition of excessive unlabeled specific competition probes (SCP) (Figure 6 A lane3 and B lane2) but not by the non specific competition probe (Non-SCP) (Figure 6 A lane4 and B lane1), suggesting that the binding between the protein and DNAs was specific. Moreover, super-shift experiments showed that anti SjTHRβ-DBD serum could partially retard shift of the bands (Figure 6 C lane2 and lane3).

### Protective immune efficacy in mice induced by rSjTHRβ-LBD

In order to evaluate the protection levels induced by rSjTHRβ-LBD, the percentage reductions in the worm burden and in the liver egg count were calculated (Table 1). Comparison of mice immunized with rSjTHRβ-LBD to PBS control group, showed a 27.52% decrease in the worm burden (P < 0.05) and a 29.50% reduction in egg count (P < 0.05) obtained. The results revealed that rSjTHRβ-LBD could induce partial protection against *S. japonicum* infection.

**Table 1:** **Comparison of protective efficacy against*S. japonic*um challenge in mice induced by SjTHRβ-LBD**

**group**	**Average worm burden**	**worm reduction rate(%)**	**Eggs/g liver**	**Liver egg reduction rate(%)**
PBS	25.25 ± 4.23	—	77,210.85 ± 15,437.34	—
206	26.13 ± 7.99	—	73,623.43 ± 12,063.51	4.65
rSjTHRβ-LBD	18.3 ± 4.58	27.52*	54,432.46 ± 5,341.33	29.50*

* indicate P value <0.05.

### Specific antibody detection

**Figure 7:**
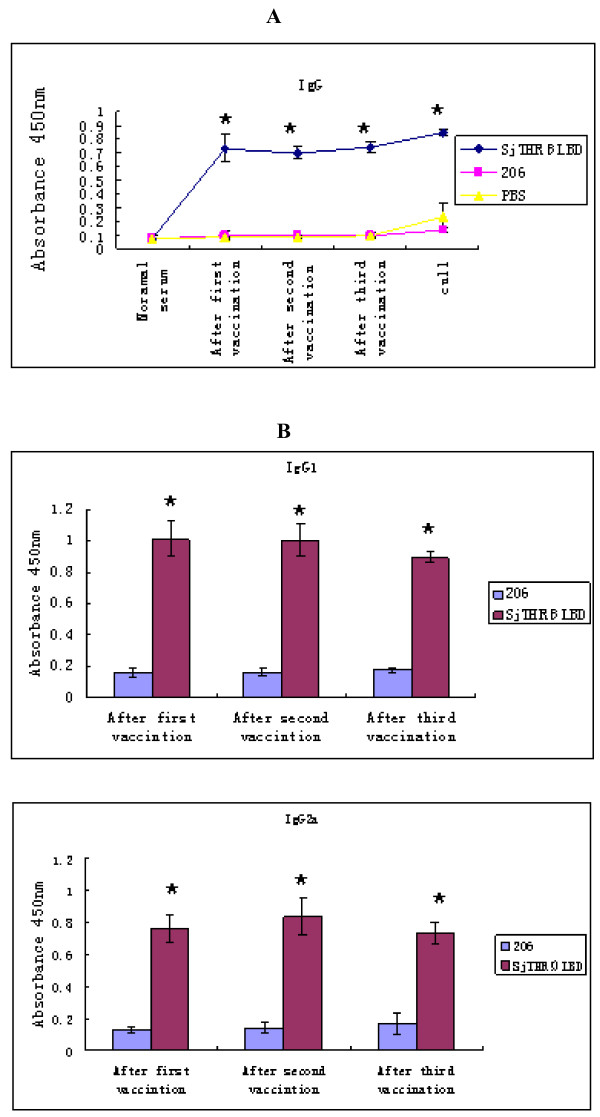
**A Detection of specific IgG antibody against rSjTHRβ-LBD by ELISA.** Mice were injected with rSjTHRβ-LBD, 206 adjuvant and PBS, respectively. Figure 7B Detection of specific IgG1 and IgG2a antibodies against rSjTHRβ-LBD. This result was obtained by three replicates with identical protocols. Vertical bars depict the ± SD about the mean. The asterisks (*) indicate significantly increased serum antibody titers compared with that of the 206 control (P < 0.01).

The level of IgG antibody specific to rSjTHRβ-LBD in the sera from both immunized and control mice as detected by ELISA is shown in Figure 7A. In the group of mice immunized with rSjTHRβ-LBD, the specific IgG antibody rose to a higher level after the first injection, thereafter it maintained a high level until the mice were culled. In addition, the level of specific IgG1 and IgG2a antibodies induced by rSjTHRβ-LBD was determined (Figure 7B). Both IgG1 and IgG2a levels increased after the first injection, and the ratio of IgG1/IgG2a did not obviously change. The specific IgG, IgG1 and IgG2a levels in both the adjuvant control group and blank control group remained at a lower level until mice were culled.

### Cytokine analysis

**Figure 8:**
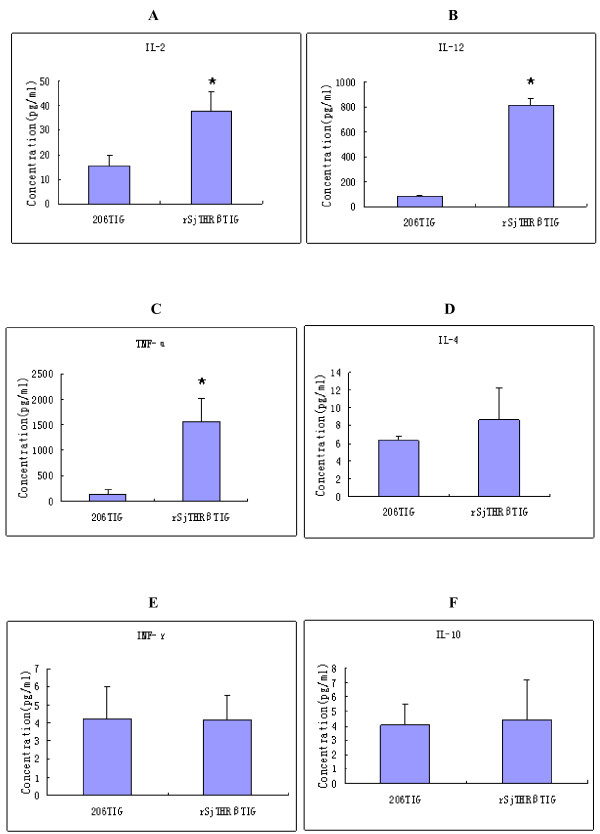
**Cytokine analysis between rSjTHRβ-LBD and 206 adjuvant group.** The level of serum IL-2 (A), IL-12 (B), TNF-α (C), IL-4 (D), INF-γ (E) and IL-10 (F) from mice immunized with rSjTHRβ-LBD plus 206 adjuvant or 206 adjuvant only. The asterisk indicates significantly increased serum cytokine level between rSjTHRβ-LBD and 206 adjuvant control groups (p < 0.05). 206TIG represents the third injection group of 206 adjuvant. rSjTHRβTIG depict the third injection group of rSjTHRβ-LBD.

IL-2, IL-4, IL-10, TNF-α, IL-12 and IFN-γ were evaluated by Bio-Plex. As shown in the Figure 8, the level of IL-2, IL-12 and TNF-α in the rSjTHRβ-LBD group were significantly higher than those in the adjuvant control group. The IL-4 and IL-10 levels in the rSjTHRβ-LBD group were slightly higher(P > 0.05) than that in the 206 adjuvant group and there was almost no difference in the IFN-γ production between the rSjTHRβ-LBD group and the 206 adjuvant group.

## Discussion

*S. japonicum* has a complex life cycle including free-living, aquatic stages and parasitic stages in the intermediate host snail or in the definitive mammalian host and is mainly responsible for intestinal and hepatosplenic schistosomiasis in China, the Philippines, and Indonesia [11]. The genomic information of *S. japonicum* suggests the existence of an integral hypothalamic–pituitary– thyroid axis in this species [17]. TH is synthesized in the thyroid gland under the control of thyroid stimulating hormone (TSH) secreted by the pituitary. TSH secretion is controlled by thyrotropin-releasing hormone (TRH), which is secreted from the hypothalamus [2]. In addition, TRH, TSH and TH perform their functions by TRH, TSH and TH receptors [17]. Among THRs, two principal THR types (THRα and THRβ) with specific physiological functions have been identified from various species, including *S. mansoni*. According to putative sequences of THR deposited in http://lifecenter.sgst.cn/schistosoma/cn/schdownload, several pairs of primer were designed to amplify SjTHRs. Based on these primers; we firstly identified a sequence encoding SjTHRβ. However, none of the primers resulted in SjTHRα sequence. The RT-PCR confirmed that SjTHRβ is constitutively expressed with higher levels from 21d and 35d worms, suggesting that SjTHRβ may have a more significant role in the developmental stages of 21d and 35d worms. The SjTHRβ contained a highly conserved DBD domain and a moderately conserved LBD domain. Our EMSA show that SjTHRβ- DBD can bind to a conserved DNA core motif AGGTCA, indicating that the function of THR is conserved during evolution. Freebern and his colleagues have proved the binding ability between SmRXR and cis-elements of the P14 gene, which is an eggshell precursor, providing evidence for a possible role in regulation of female-specific gene expression [18]. THR DNA binding is involved in both positive and negative gene regulation [19]. Further identification of the THRE in specific target gene in *S. japonicum* will help to understand the regulation mechanism of THR.

Even though praziquantel is highly effective in curing schistosomiasis, it still has its limitations that cannot prevent high frequency of re-infection and repair injury [20]. Thus, the discovery of an effective vaccine remains the most potentially powerful means of control for this disease. Previous studies have described the cross-talk of host-parasite relationships between endocrine and immune mediators [21]. The influence of TH on the immune system is well characterized and could mediate its action on infection. Saule et al. showed that the variation of host TH and interleukin-7 could favor the development of *S. mansoni*[22]. Considering that *S. japonicum* might have an integral hypothalamic–pituitary–thyroid axis in its genome, we speculate that THRβ of *S. japonicum* could bind to ligand (TH) of both itself and its host. TH might act through THR, a member of the nuclear receptor superfamily I that intervenes in the expression of numerous genes, on both host and parasite. So, we assumed that SjTHR might have a role in immune regulation during the *S. japonicum* infection process. Here, we cloned and over-expressed SjTHRβ-LBD to evaluate its protective efficacy against schistosoma. In this experiment, rSjTHRβ-LBD could induce partial levels of protection against *S. japonicum* challenge. The mice vaccinated with rSjTHRβ-LBD elicited high levels of specific IgG as well as its subtypes IgG1 and IgG2a antibody via ELISA. IgG2a might predominantly activate T helper 1 cells, while IgG1 antibody mainly differentiated Th0 precursor in the Th2 direction in mice [23]. Taken together, antibody profiles of vaccinated mice revealed the protective response to be a mixed Th1/Th2 types. Cytokines acting on lymphocytes play an important regulatory and signaling role in the development of an immune response. It is well known that IL-12, together with TNF-α, IL-2 and IFN-γ have long been classified as a type 1 immune cytokines [24,25]. The presence of IL-2, IFN-γ, and IL-12 activates the Th0 precursor cells to become Th1 inflammatory T cells. IL-12 gene polarizes the immune responses towards Th-1 cell development and stimulates the strongest CTL activity [26,27]. In addition, TNF-α is a pro-inflammatory cytokine involved in the cytokine cascade and leukocyte recruitment. On the other hand, IL-4 as well as IL-10 induces a Th0 precursor becoming an armed Th2 helper cell [26]. IL-4 is a prominent Th2 cytokine that plays an important part in stimulating humoral immune responses [28]. Given that the cytokines IL-2, IL-12 and TNF-α in the SjTHRβ-LBD group were significantly enhanced, whereas, the levels of serum IL-4 and IL-10 in the rSjTHRβ-LBD group and 206 adjuvant group were not obviously different, we suggested that rSjTHRβ-LBD can induce considerably higher levels of Th-1 response enhancing cytokines (IL-2, IL-12 and TNF-α) than Th-2 response enhancing cytokines (IL-10, IL-4). rSjTHRβ- LBD vaccination could stimulate a mixed Th1/Th2 type with Th1 dominant in humoral and T cell responses.

## Conclusion

Herein, we identified SjTHRβ as the first member of NR superfamily in *S. japonicum.* Multiple sequence alignment and constructed evolutionary tree showed that SjTHRβ belongs to nuclear superfamily I. EMSA analysis showed that SjTHRβ-DBD could bind to a conserved DNA core motif, suggesting SjTHRβ might play an important role in gene regulation in *S. japonicum*. In addition, immunization of BALB/c mice with rSjTHRβ-LBD vaccination could induce partial protective efficacy against schistosome infection, indicating SjTHRβ might be a vaccine candidate for schistosomiasis. A full understanding of the role of SjTHRβ in the biology of *S. japonicum* will add to our knowledge of nuclear receptor gene regulation in schistosomes. Ongoing studies to identify more nuclear receptors involved in host-parasite interactions will aid in mechanistic studies to determine useful drug targets to modulate parasite or host biochemical pathways [29] and treat schistosomiasis.

## Competing interests

The authors declare that they have no competing interests.

## Authors’ contributions

Conceived and designed the experiments: CHQ,JJL. Performed the experiments: CHQ, MMW, DZA. Analyzed the data: CHQ. Wrote the paper: CHQ. JJL, CHQ, SFL, YH, ZQF revised the manuscript. All authors read and approved the final manuscript.
